# Gossypetin Is a Novel Modulator of Inflammatory Cytokine Production and a Suppressor of Osteosarcoma Cell Growth

**DOI:** 10.3390/antiox12091744

**Published:** 2023-09-10

**Authors:** Carina Proença, Ana Teresa Rufino, Isabela Santos, Hélio M. T. Albuquerque, Artur M. S. Silva, Eduarda Fernandes, José Miguel P. Ferreira de Oliveira

**Affiliations:** 1 LAQV, REQUIMTE, Laboratory of Applied Chemistry, Department of Chemical Sciences, Faculty of Pharmacy, University of Porto, 4050-313 Porto, Portugal; cproenca@ff.up.pt (C.P.); arufino@ff.up.pt (A.T.R.); up202112015@edu.ff.up.pt (I.S.); egracas@ff.up.pt (E.F.); 2 LAQV, REQUIMTE, Department of Chemistry, Campus Universitario de Santiago, University of Aveiro, 3810-193 Aveiro, Portugal; helio.albuquerque@ua.pt (H.M.T.A.); artur.silva@ua.pt (A.M.S.S.)

**Keywords:** bone sarcoma, chemotherapeutic agents, flavonoid, 3,3′,4′,5,7,8-hexahydroxyflavone, apoptosis, proinflammatory cytokines

## Abstract

Osteosarcoma (OS) is a common childhood sarcoma, and its treatment is hindered by adverse effects, chemoresistance, and recurrence. Interleukin (IL)-6 production by tumors plays a significant role in inflammation, carcinogenesis, and metastasis. This study aimed to investigate the antiproliferative potential of luteolin derivatives in OS and to evaluate interleukin production. MG-63, Saos-2, HOS, and 143B human OS cell lines were incubated with luteolin and eight derivatives containing hydroxy, chlorine, or alkyl substitutions. The cell viability and growth were evaluated in the presence of these compounds. Apoptosis was also examined through the analysis of the Bax expression and caspase-3 activity. Finally, the gossypetin effects were measured regarding the production of proinflammatory cytokines interleukin (IL)-6, IL-1β, and IL-12p70. Our findings show that gossypetin was the most potent compound, with proliferation-suppressing activities that induced a series of critical events, including the inhibition of the cell viability and growth. Apoptosis was associated with enhanced caspase-3 activity and increased Bax expression, indicating the involvement of the intrinsic pathway of apoptosis. Moreover, pre-/co-treatment with gossypetin significantly reduced the autocrine production of proinflammatory cytokines. Further investigation is required; nevertheless, considering the link between inflammation, carcinogenesis, and metastasis in OS, our findings suggest that gossypetin exhibits anti-proliferative and anti-inflammatory properties that are potentially relevant in the clinical context.

## 1. Introduction

Osteosarcoma (OS) is a mesenchymal bone cancer that comprises highly heterogeneous subtypes [1]. It shows an incidence peak in adolescence and has a high potential for local invasion and early lung metastasis [2,3]. The standard of care includes methotrexate, doxorubicin, and cisplatin, and the major challenge is the development of chemo-resistance and metastasis [3,4]. Since the 1980s, OS therapies have not experienced major improvements and are limited by an overall survival of approximately 20–30% in the event of metastasis [5].

As is the case in other cancer types, OS cells can become insensitive to apoptosis [6,7,8]. Enhancing apoptosis is therefore a valuable strategy for overcoming OS proliferation and chemoresistance [9,10]. Several drugs have been tested in clinical trials, including inhibitors of tyrosine kinases, cyclin-dependent kinases, Poly (ADP-ribose) polymerase (PARP), and mammalian target of rapamycin (mTOR) [4,11]. Despite ongoing trials being conducted to evaluate the multi-kinase inhibitors regorafenib and sorafenib, the long-term survival remains unchanged [12].

Chronic inflammation is a known driver of cancer initiation, progression, chemoresistance, and metastasis [13]. During late osteoblastogenesis, the multifunctional cytokine transforming growth factor (TGF)-β inhibits mesenchymal stem cell (MSC) transdifferentiation into osteoblasts. OS cells, on the other hand, release receptor activator of nuclear factor κ-Β ligand (RANKL) and inflammatory interleukin (IL)-6, which stimulate bone resorption and further production of TGF-β [14]. In subjects with OS, the serum levels of interleukin 1 receptor antagonist (IL-1Ra), IL-6, and IL-8 are found to be increased compared to healthy subjects [15]. Importantly, IL-6 has been identified as a mediator of OS lung tropism [16]. IL-6 produced by MSCs in the bone microenvironment promote metastization via the signal transducer and activator of transcription 3 (STAT3) pathway [17]. Importantly, the production of IL-8 was found to form loops between OS and MSCs [18]. More recently, it was demonstrated that doxorubicin induced the autocrine production of proinflammatory IL-6 and IL-1β in OS cells [19]. It is worth noting that in OS, IL-1β is responsible for the increased expression of programmed cell death ligand 1 (PD-L1), which is associated with cancer cell evasion, highlighting the role of IL-1β in tumor relapse [19].

Plant polyphenols protect against diseases such as obesity, diabetes, gastrointestinal disorders, and cancer while showing positive effects on osteoblast/osteoclast interaction [20,21,22,23]. Paradigmatically, the flavonoid luteolin was shown to affect human bone via the stimulation of alkaline phosphatase activity, the induction of bone differentiation, the suppression of osteoclastogenesis induced by RANKL and macrophage colony-stimulating factor (M-CSF), and the inhibition of proinflammatory cytokine production [10,24,25,26]. Previously, Dwi Antika et al. reported that gossypetin (3,8-dihydroxyluteolin) possessed osteoprotective activity and attenuated RANKL-induced osteoclast formation [27]. In addition, this compound did not reveal toxicity effects to the human keratinocyte HaCaT cell line exposed to UV irradiation, whilst preventing the in vitro and in vivo activation of the p38 and ERK pathways implicated in UV-induced basal cell carcinoma [28]. In vivo, Khan and co-workers assessed the protective effects of gossypetin against the DNA damage caused by γ-radiation [29]. Freshly isolated hepatocytes from Swiss albino mice were incubated with gossypetin for 1 h, followed or not by irradiation (5 Gy, γ-radiation). In the absence of irradiation treatment, the gossypetin treatments (20 and 40 µmol/L) did not result in statistically significant different parameters of genotoxicity compared to the control, as shown using the comet assay. Importantly, pre-/co-incubation and irradiation significantly reduced the damage caused by radiation in a concentration-dependent manner. Under the same experimental conditions (incubation with gossypetin and irradiation), freshly isolated mice hepatocytes were incubated with the fluorescent probe 2′,7′ -dichlorofluorescin diacetate (DCFDA) [29]. In agreement with the comet assay results, the authors observed that incubation with gossypetin did not result in a measurable increase in intracellular reactive oxygen species (ROS). Moreover, pre/co-incubation with gossypetin attenuated, in a concentration-dependent manner, the ROS production induced by the irradiation treatment.

**Figure 1:**
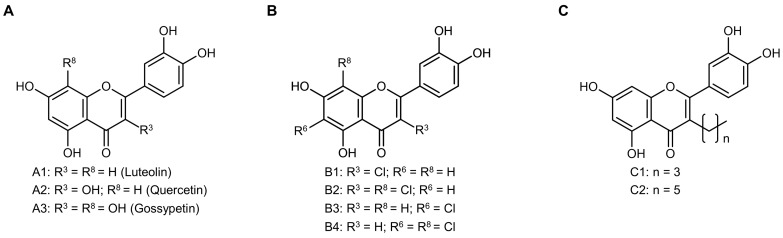
Chemical structures of the studied flavonoids. (**A**) hydroxylated; (**B**) chlorinated; and (**C**) alkylated flavonoids.

Considering its anti-inflammatory role, it is hypothesized that luteolin or structurally similar compounds present relevant anti-inflammatory activity that can be explored in the context of OS therapy. The present work aimed to evaluate the cell viability inhibitory activity of luteolin and a group of eight derivatives in OS cells and to investigate the cytotoxic and anti-inflammatory properties of the most active compound. To achieve this, the OS cell lines MG-63, Saos-2, HOS, and 143B were incubated with luteolin and derivatives with hydroxy, chlorine, and alkyl substitutions at C-3, C-6, and/or C-8 (Figure 1).

The most active inhibitor of OS viability, compound **A3** (gossypetin), was subsequently evaluated for its apoptotic and anti-inflammatory effects.

## 2. Materials and Methods

### 2.1. Cell Culture Media and Reagents

Dulbecco’s modified Eagle’s medium (DMEM), fetal bovine serum (FBS), L-glutamine, penicillin-streptomycin, and trypsin-ethylenediamine tetraacetic acid (0.25% trypsin, 1 mM EDTA) were purchased from Thermo Fisher Scientific (Carlsbad, CA, USA). The following reagents were obtained from Sigma-Aldrich (St. Louis, MO, USA): accutase solution, dimethyl sulfoxide (DMSO), Dulbecco’s phosphate-buffered saline (DPBS), staurosporine, sulforhodamine B (SRB), trichloroacetic acid (TCA), trizma base, mouse monoclonal anti-β-actin antibody, and compounds luteolin (**A1**) and quercetin (**A2**). Gossypetin (**A3**) was purchased from Indofine Chemical Company (Hillsborough, NJ, USA). The following flavonoids were obtained through synthesis according to previous papers: 3′,4′,5,7-tetrahydroxy-3-chloroflavone (**B1**), 3′,4′,5,7-tetrahydroxy-6-chloroflavone (**B2**), 3′,4′,5,7-tetrahydroxy-3,8-dichloroflavone (**B3**), 3′,4′,5,7-tetrahydroxy-6,8-dichloroflavone (**B4**), 3-butyl-3′,4′,5,7-tetrahydroxyflavone (**C1**), 3-hexyl-3′,4′,5,7-tetrahydroxyflavone (**C2**) [30,31]. Antibodies against Bax (Cat. No. 5023S) and β-actin (Cat. No. 5441) were purchased from Cell Signaling Technology (Danvers, MA, USA) and Sigma-Aldrich (St. Louis, MO, USA), respectively. Horseradish peroxidase (HRP)-conjugated secondary antibodies to anti-rabbit (Sigma, Cat. No. 12-348) and anti-mouse (Santa Cruz Biotechnology, Cat. No. sc-516102) were obtained from Sigma-Aldrich (St. Louis, MO, USA) and Santa Cruz Biotechnology (Santa Cruz, TE, USA), respectively.

### 2.2. Cell Culture and Exposure Conditions

The OS cell lines used in this study present diverse morphologies. The morphology of MG-63 cells is fibroblastic, whilst the morphology of Saos-2 cells is epithelial. HOS and 143B cells display a mixed morphology. The 143B cells were derived from the HOS cell line; however, they are thymidine-kinase deficient and possess heterozygous KRAS mutations (p.G12S/p.A59T) [32]. The karyotype of these cell lines shows variation in the chromosome number, ranging from hypotriploid (MG-63, Saos-2) to hyperdiploid (HOS, 143B) (ECACC). Disruption of p53 expression is frequently associated with apoptosis resistance. The p53 tumor suppressor protein, encoded by the *TP53* gene, is distinctly expressed among the OS cell lines described in this study. MG-63 cells possess an intact *TP53* exonic sequence; however, the gene is highly methylated and contains rearrangements in intron 1, rendering this cell line p53-deficient [33,34,35,36,37]. In Saos-2, a deletion in exons 4–8 of *TP53* also results in a null mutant [33,34,35,38,39]. In 143B cells and the parental cell line, HOS, p53 is strongly expressed as a mutant protein, p.R516P [32,33,34,35,36,38,39,40].

Disruption of bone remodeling is a primary attribute of osteosarcomas. Osteosarcoma cell invasion significantly disturbs the equilibrium between bone breakdown and formation. This disturbance causes the release of cytokines or growth factors within the bone structure, including transforming growth factor-β (TGF-β), and these factors foster OS progression. Importantly, among the OS cell lines of this study, the MG-63 cell line can be highlighted due to its elevated expression of both TGF- β receptors I and II, setting this cell line as a relevant model in studies related to cancer cell proliferation and autocrine cytokine production [41].

The human OS cell lines MG-63 (ECACC 86051601), Saos-2 (ECACC 89050205), HOS (ECACC 87070202), and 143B (ECACC 91112502) and the human fetal lung fibroblast cell line MRC-5 (ECACC 05090501) were purchased from the European Collection of Authenticated Cell Cultures (ECACC, Salisbury, UK) in September 2020 and authenticated through Short-Tandem Repeat (STR) profiling. Upon arrival, the cells were cryopreserved from 3 subsequent passages. After resuscitation, the cells were passaged for less than 4 months (maximum passage number 40 for OS cells and 30 for MRC-5 human lung fibroblasts). The cells were routinely tested for the presence of mycoplasma. The cells were maintained in DMEM supplemented with 10% FBS, 2 mM L-glutamine, 100 U/mL penicillin, and 100 µg/mL streptomycin. They were then incubated at 37 °C with 5% CO_2_ in a humidified atmosphere. The cells were subcultured through detachment with 0.25% trypsin—1 mM EDTA. Flavonoid stock solutions were prepared to a concentration of 100 mmol/L in DMSO. For all conditions, including DMSO solvent control, the DMSO concentration was 0.1% in complete DMEM. The flavonoid concentrations and the incubation period used in the cytotoxicity assays were based on previous studies in OS cell lines incubated with flavonoids of a similar chemical structure [42,43]. For the subsequent assays, the cells were incubated with flavonoids at concentrations below the half-maximal inhibitory concentration (IC_50_) values determined in the cytotoxicity assays. The population doubling of MG-63 was ~30 h under standard growth conditions. Therefore, for the subsequent assays, the time point of 24 h was chosen as the longest time point for incubation with the flavonoid gossypetin in order to dismiss the confounding factor of proliferation in the assessment of the percentage of apoptotic cells.

### 2.3. Cytotoxicity Assays

The OS (5 × 10^3^ cells/well) cell lines and the normal lung MRC-5 (1 × 10^4^ cells/well) cell line were plated in 96-well plates. After adhesion, the cells were treated with flavonoid compounds—10, 20, 40, 80, and 160 μmol/L—for 48 h. The WST-8 [2-(2-methoxy-4-nitrophenyl)-3-(4-nitrophenyl)-5-(2,4-disulfophenyl)-2H-tetrazolium, monosodium salt] assay was performed using the Cell Counting Kit-8 (CCK-8) (Sigma-Aldrich, St. Louis, MO, USA). Briefly, after incubation with flavonoid compounds, the medium was replaced with complete medium containing 1:10 (*v*/*v*) CCK-8 reagent and the cells were incubated for 2 h at 37 °C. After incubation, the absorbance was measured at 450 nm in a Synergy HT Multi-mode Microplate Reader (BioTek Instruments, Winooski, VT, USA). For the SRB assay, after exposure, the cells were fixed for 1 h with 10% (*w*/*v*) TCA at 4 °C. Subsequently, after microplate washing with water and drying, the cells were incubated for 30 min with SRB solution (0.05% *w*/*v* in 1% acetic acid). Then, the SRB reagent was removed and the stained cells were washed four times with 1% acetic acid (*v*/*v*). After drying, 10 mM tris-base (unbuffered, pH~10.5) was added, followed by shaking for 10 min. The absorbance at 510 nm was measured in a Synergy HT Multi-mode Microplate Reader (BioTek Instruments).

### 2.4. Apoptosis Detection

For the quantitative assessment of apoptosis, 1 × 10^5^ MG-63 cells/well were seeded in 6-well plates and incubated overnight at 37 °C. After adhesion, the cells were incubated with gossypetin—10, 20, and 40 µmol/L—for 24 h. After incubation, the culture medium from each condition was collected and the attached cells were washed briefly with DPBS. Cells were harvested after detachment with Accutase reagent. For each condition, the culture medium and detached cells were pooled and centrifuged at 200× *g* for 4 min. For this step, the FITC Annexin V Apoptosis Detection Kit I (BD Pharmingen, San Diego, CA, USA) was used according to the manufacturer’s instructions, including washing with DPBS and staining with fluorescein isothiocyanate (FITC)-annexin V and propidium iodide (PI). The samples were analyzed in a BD Accuri C6 instrument (BD Biosciences, San Jose, CA, USA), with laser excitation at 488 nm. At least 10,000 gated events were analyzed in the FL1 and FL3 channels. Population percentages were calculated from the number of cells in each quadrant divided by the total number of cells using the BD Accuri C6 software (version 264.21).

### 2.5. Western Blot Analysis

Bax expression was determined in MG-63 cells. For this, 5 × 10^5^ MG-63 cells were seeded in 6-well plates and incubated overnight at 37 °C. After adhesion, the cells were exposed to gossypetin—20 and 40 µmol/L—for 24 h. The cells were washed 2 times with cold DPBS and then scraped in 150 µL Radio-Immunoprecipitation Assay (RIPA) buffer (pH 7.0) containing protease inhibitors. The cells were stored at −80 °C until required. Cell lysates were centrifuged at 200× *g* for 5 min at 4 °C and the supernatant was used for the assay. The protein concentration was determined using the Bradford method and cell lysates were denatured in sample buffer [0.5 M tris-HCl, pH = 6.8, 2% (*v*/*v*) sodium dodecyl sulphate (SDS), 10% glycerol, 25% (*v*/*v*) 2-mercaptoethanol and bromophenol blue]. The protein samples (20 µg each) were loaded and separated through electrophoresis on 10% (*v*/*v*) SDS-PAGE followed by western blotting (160 V, 1 h), and subsequently transferred to polyvinylidene fluoride (PVDF) membranes through wet transference (3 h, 400 mA). To examine the protein levels, PVDF membranes were incubated overnight at 4 °C with a primary anti-human monoclonal antibody against Bax (1:1000). Mouse anti-human actin monoclonal antibody was used to detect actin as a loading control. Immune complexes were detected with the enhanced chemiluminescent (ECL) reagent, and the bands were analyzed using the CLIQS Gel Image Analysis Software (version 7.0) from TotalLab (Newcastle, UK). The results were normalized by calculating the ratio between the intensities of the bands corresponding to the protein of interest and the protein used as a loading control.

### 2.6. Caspase Activity

For the determination of caspase activity, 5 × 10^5^ MG-63 cells were seeded in 25 cm^2^ culture vessels and incubated overnight at 37 °C. After adhesion, the cells in each vessel were exposed to gossypetin—20 and 40 µmol/L—for 14 h and 24 h. Following exposure, the medium was collected from each condition and the attached cells were washed with DPBS and harvested after detachment with Accutase reagent. The culture medium and detached cells from each condition were pooled and centrifuged at 200× *g* for 4 min. After this step, the Caspase-3/CPP32 Colorimetric Assay Kit (Biovision, Palo Alto, CA, USA) was used, following the manufacturer’s instructions. Briefly, after careful resuspension and incubation in lysis buffer, the cell lysates were centrifuged, and the corresponding supernatants were transferred to new tubes. The protein concentration was determined using the Bradford method. From each supernatant, 200 µg total protein were added to 96-well plates. The samples were diluted in reaction buffer and 200 µmol/L DEVD-pNA substrate was added. After 2 h incubation at 37 °C, the absorbance was measured at 405 nm in a Synergy HT Multi-mode Microplate Reader (BioTek Instruments).

### 2.7. Cytokine Production

Cytokine production was evaluated in MG-63 cells. For this, 1.5 × 10^5^ cells/well were seeded in 24-well plates. After cell adhesion, the medium was replaced with complete DMEM containing 40 µmol/L gossypetin or solvent control. After incubation at 37 °C for 1.5 h, a concentrated cytokine mixture, containing Tumor Necrosis Factor (TNF), IL-1β, and interferon-γ (IFN-γ), was added to the medium, to a final concentration of 10 ng/mL for each cytokine. Alternatively, complete medium was added. The cells were incubated for 24 h at 37 °C; subsequently, cell culture supernatants were harvested from each condition and processed according to the instructions from the BD Cytometric Bead Array (CBA) Human Inflammatory Cytokine Kit (BD Biosciences, Sans Jose, CA, USA). In brief, mixed capture beads were mixed with Human Inflammatory Cytokines Phycoerythrin (PE) Detection Reagent and with culture supernatant (test samples) or with cytokine standard mixture in serial 10-fold dilutions (standard samples). After incubation for 3 h in the dark, wash buffer was used to wash the samples and resuspend the bead pellets. The samples were analyzed in a BD Accuri C6 instrument (BD Biosciences, San Jose, CA, USA). The BD™ CellQuest Software (version 5.2.1) was used with the CBA Instrument Setup template. The following parameters were determined for bead compensation and data acquisition: FSC, SSC, FL1, FL2, and FL3, according to the BD CBA Human Inflammatory Cytokine Kit instructions. The assayed cytokines were distinguished with the FL3 height parameter, while quantification was determined from the FL2 height parameter. Analysis of the flow cytometric data for cytokine quantification was accomplished using the FCAP Array software (version 3.0).

### 2.8. Statistical Analysis

At least three independent experiments were performed per assay (*n* ≥ 3). The IC_50_ values were determined using the GraphPad Prism software (version 6.0) (San Diego, CA, USA), using the nonlinear dose-response inhibition model. Results are mean ± standard error of the mean (SEM). GraphPad Prism was used for graphical representation and to compare control vs. experimental groups through one-way analysis of variance (ANOVA) with the post-hoc Dunnett’s test. For the cytokine production experiment, groups were compared through one-way analysis of variance (ANOVA), followed by the post-hoc Holm-Šidák multiple comparison test. For all comparisons, differences were considered statistically significant for significance levels of * *p* < 0.05, ** *p* < 0.01, *** *p* < 0.001, and **** *p* < 0.0001.

## 3. Results

### 3.1. Structure-Activity Relationship (SAR)

**Figure 2:**
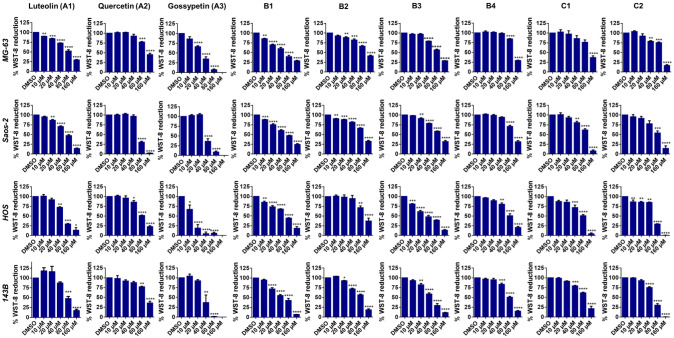
Inhibition of OS cell viability. MG-63, Saos-2, HOS and 143B human OS cell lines were incubated with flavonoids (0–160 µmol/L) for 48 h. Subsequently, WST-8 viability assays were conducted. Each row indicates the cell line used (left) and each column indicates the compound tested (top). The data are mean ± SEM (*n* ≥ 3). Significant differences (asterisks) are shown relative to 0.1% DMSO control; * *p* < 0.05, ** *p* < 0.01, *** *p* < 0.001, and **** *p* < 0.0001 (one-way ANOVA).

**Figure 3:**
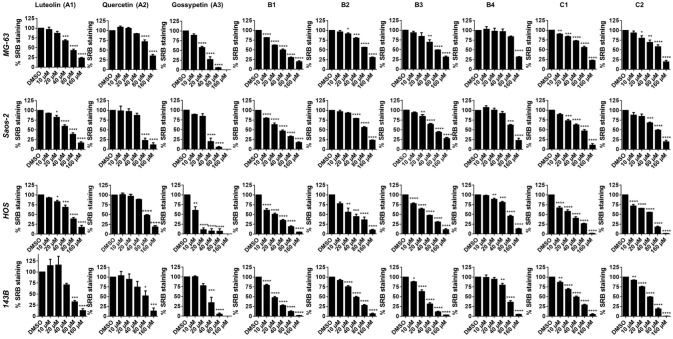
Inhibition of OS cell growth. MG-63, Saos-2, HOS and 143B human OS cell lines were incubated with flavonoids (0–160 µmol/L) for 48 h. Subsequently, SRB growth assays were conducted. Each row indicates the cell line used (left) and each column indicates the compound tested (top). The data are mean ± SEM (*n* ≥ 3). Significant differences (asterisks) are shown relative to 0.1% DMSO control; * *p* < 0.05, ** *p* < 0.01, *** *p* < 0.001, and **** *p* < 0.0001 (one-way ANOVA).

After incubation with flavonoid compounds for 48 h, the IC_50_ values were determined for each OS cell line (Table 1). Overall, the inhibition of cell viability and growth by the flavonoid compounds was stronger in the HOS and 143B cell lines compared to the MG-63 and Saos-2 cells. The presence of the 8-hydroxy group (compound **A3**) was associated with the lowest IC_50_ values among all the compounds. The presence of 3-chlorine (**B1** versus **A1**) resulted in the enhanced inhibition of cell growth, according to the results obtained in the SRB assay. The concentration-dependent inhibition of OS cell viability and growth was observed for all of the compounds (Table 1; Figure 2 and Figure 3).

Gossypetin (compound **A3**) was the most potent inhibitor of OS cell viability and growth in vitro (Table 1). Under the culture conditions of the study, HOS cells were the most sensitive to gossypetin, followed by MG-63, 143B and Saos-2 cells. Representative micrographs of all data are provided as online dataset [44]. Conversely, normal lung fibroblasts were the least affected by gossypetin, showing significant decrease in cell viability and growth at the concentrations of 80 and 160 µmol/L (Figure S1).

### 3.2. Gossypetin Induces the Intrinsic Apoptotic Pathway

**Figure 4:**
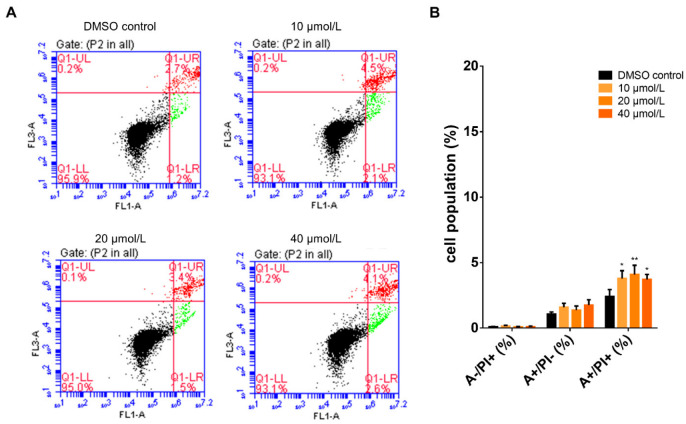
Influence of gossypetin on the percentage of apoptotic MG-63 cells. After 24 h incubation with gossypetin, MG-63 cells were harvested, incubated with FITC-annexin V and PI, and analyzed by flow cytometry. (**A**) representative dot plot diagrams of MG-63 cells labelled with FITC-annexin V (FL1) and PI (FL3); (**B**) percentage of cells identified as FITC-annexin-V/PI: +/− (early apoptotic), +/+ (late apoptotic + necrotic) and −/+ (necrotic). The data are mean ± SEM (*n* ≥ 3). Significant differences (asterisks) are shown relative to 0.1% DMSO control; * *p* < 0.05, ** *p* < 0.01 (one-way ANOVA).

The MG-63 cells were incubated with gossypetin (0–40 µmol/L) for 24 h and the percentage of apoptotic cells was determined using the Annexin-V/PI assay (Figure 4).

At the tested concentrations (10, 20, and 40 µmol/L), gossypetin increased the percentage of apoptotic cells, as evidenced by the increased percentage of FITC-annexin-V labelled cells.

**Figure 5:**
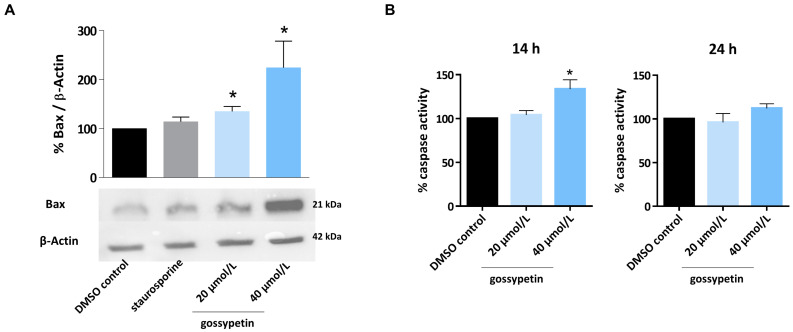
Influence of gossypetin on Bax expression and caspase activity. (**A**) Detection of proapoptotic Bax by Western blot. MG-63 cells were incubated with gossypetin (20 and 40 µmol/L) for 24 h, and protein expression was determined by Western blot. Actin was used as loading control. (**B**) Determination of caspase 3 activity. MG-63 cells were incubated with gossypetin (20 and 40 µmol/L) for 14 h and 24 h, and caspase 3 activity was determined by colorimetric assay with DEVD-pNA substrate. The data are mean ± SEM (*n* ≥ 3). Significant differences (asterisks) are shown relative to 0.1% DMSO control; * *p* < 0.05 (one-way ANOVA).

To investigate the apoptotic mechanism, the MG-63 cells were incubated with gossypetin and compared to the solvent control. Subsequently, the expression of proapoptotic Bax protein was determined, together with the caspase 3 activity (Figure 5).

The presence of gossypetin increased the expression of the proapoptotic protein Bax (Figure 5A), indicating the activation of the intrinsic pathway of apoptosis. The caspase 3 activity (Figure 5B) was increased by the gossypetin treatment, with a significant increase in the caspase 3 activity observed after 14 h incubation with 40 µmol/L gossypetin.

### 3.3. Under Proinflammatory Conditions, Gossypetin Alters Cytokine Production in OS Cells

**Figure 6:**
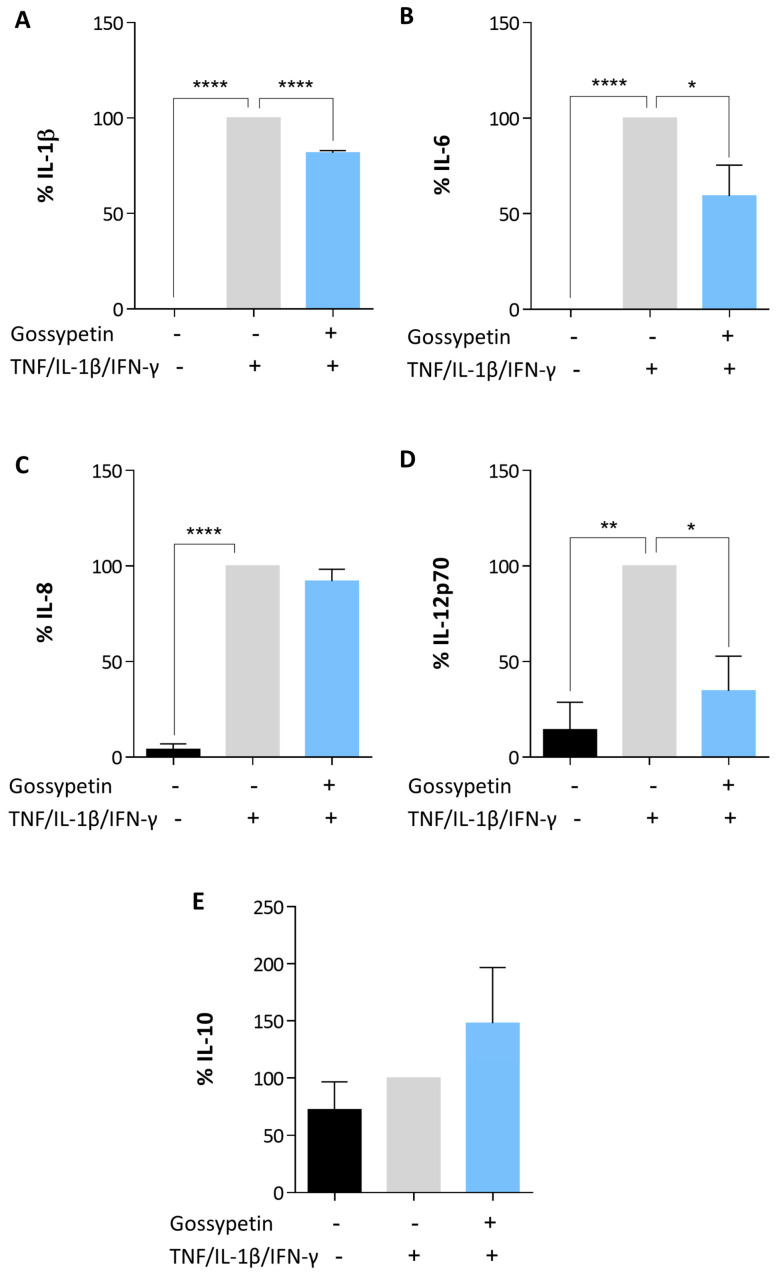
Influence of gossypetin on cytokine production. MG-63 cells were pre-incubated with 40 µmol/L gossypetin for 1.5 h and additionally incubated with a proinflammatory cytokine mixture (10 ng/mL TNF, IL-1β and IFN-γ) for 24 h. Cytokine production was determined from the culture supernatants, and normalized to control condition (proinflammatory cytokine mixture treatment only). Relative cytokine levels refer to the cytokines: (**A**) IL-1β; (**B**) IL-6; (**C**) IL-8; (**D**) IL-12p70; (**E**) IL-10. The data are mean ± SEM (*n* ≥ 3). Significant differences (asterisks) are shown relative to 0.1% DMSO control; * *p* < 0.05, ** *p* < 0.01, and **** *p* < 0.0001 (one-way ANOVA).

Cytokine production was investigated in the MG-63 cells pre-incubated with gossypetin and additionally incubated with a proinflammatory cytokine mixture. The cytokine levels in the cells preincubated with gossypetin followed by co-incubation with the solvent control were identical to the cytokine levels in the cells from the solvent control, showing that under the conditions of the study, gossypetin alone did not alter the production of the selected cytokines in the absence of a proinflammatory stimulus. In the presence of a proinflammatory stimulus, in the MG-63 cells pre-incubated with gossypetin, a decrease was observed in the production of proinflammatory cytokines IL-1β, IL-6, and IL-12p70 (Figure 6).

The IL-8 production was not significantly affected by the gossypetin treatment. Regarding the production of IL-10, even though statistical significance was not reached, a tendency toward increased levels of this cytokine was observed in the cells pre-incubated with gossypetin.

## 4. Discussion

Recent studies have provided significant insight into the role of inflammation in OS progression, including the involvement of the STAT3/COX-2 axis in OS metastasis [45,46]. These studies established the negative impact of systemic inflammatory markers on overall survival and disease-free survival in OS patients. Therefore, the identification of compounds with anti-inflammatory properties relevant to OS inhibition represents an additional therapeutic opportunity for this disease. Following this rationale, we previously identified flavonoid compounds including luteolin, chlorinated flavonoids, and 3-alkylflavones as potent antioxidant and anti-inflammatory agents in acellular models, human blood cells, and whole blood [31,47,48]. These studies highlighted the important features of these groups of compounds, such as the scavenging of H_2_O_2_ and HOCl, the reduction in ferric ion, the inhibition of the COX-2-mediated production of prostaglandin E_2_, and the inhibition of proinflammatory cytokine production. Nevertheless, the potential modulatory effects of luteolin and derivatives on cytokine production and inflammation in OS remained undisclosed.

**Figure 7:**
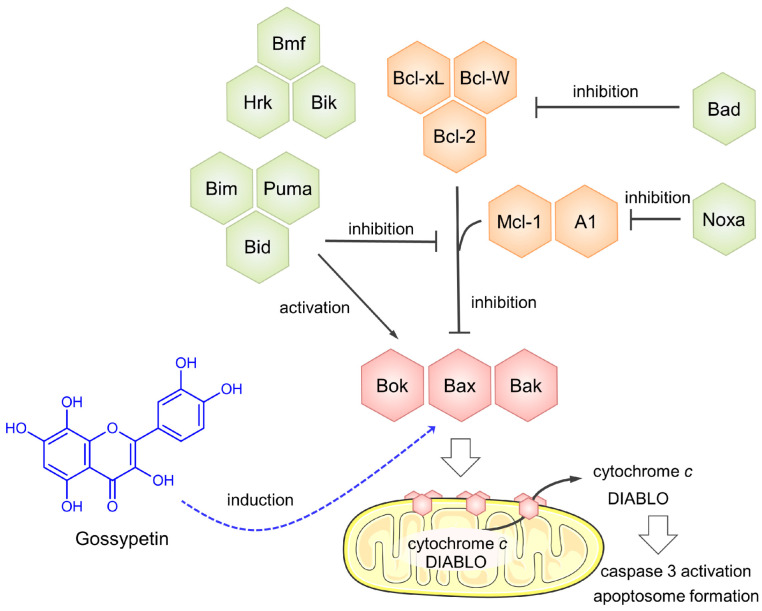
Mechanism of apoptotic action of gossypetin in MG-63 cells. Gossypetin induces the expression of BAX protein. In turn, this pro-apoptotic protein provokes mitochondrial outer membrane permeabilization, with consequential release of cytochrome c and Smac/DIABLO proteins, responsible for caspase 3 activation and apoptosome.

Among the compounds tested in the present study, luteolin and quercetin were previously reported to inhibit OS cell proliferation [49,50,51,52,53,54,55,56,57,58,59]. In MG-63 cells, luteolin (44 µmol/L, 24 h) and quercetin (40 µmol/L, 48 h) induce apoptosis, which is associated with increased cleaved caspase 3 and Bax expression and decreased Bcl-2 expression, suggesting the activation of the intrinsic pathway of apoptosis [50,56]. Moreover, in the U2OS osteosarcoma cell line, luteolin (100 µmol/L, 24 h) induces apoptosis and was reported to decrease the mitochondrial membrane potential, highlighting the activation of the intrinsic pathway [49]. In this cell line, incubation with quercetin (50 µmol/L, 24 h; and 100 µmol/L, 48 h) also induced apoptosis via the intrinsic pathway, and provoked a decrease in cell migration, linked to the inhibition of JAK2 activity and STAT3 phosphorylation [53,55,57]. Additionally, autophagy was induced by luteolin (100 µmol/L, 24 h) in osteosarcoma U2OS cells and by quercetin (100 µmol/L, 24 h) in MG-63 cells [49,58]. Among the flavonoid compounds tested in this study, gossypetin emerged as a novel and highly potent inhibitor of OS cell viability and growth. Notably, there were no prior reports on the effects of gossypetin on cell viability and growth in osteosarcoma. This flavonoid, notwithstanding, was described to inhibit cancer cell proliferation in vitro and in vivo in prostate and esophageal cancer models [60,61]. In the present study, gossypetin—20 and 40 µmol/L for 24 h—induced the expression of proapoptotic Bax protein in MG-63 cells, indicating the involvement of the intrinsic pathway of apoptosis. This observation is in line with a study by Lee et al., in which incubation with gossypetin—25, 50, and 100 µmol/L for 24 h—increased Bax expression in the androgen-insensitive DU145 prostate cancer cell line [61]. Increased Bax expression was also observed by Xie et al. in esophageal cancer cells upon incubation with gossypetin—20 and 40 µmol/L for 24 h [60]. The mechanism of action of gossypetin in the apoptotic process in MG-63 is depicted in Figure 7.

Further cancer cell death pathways were reported for gossypetin. For example, in the androgen-sensitive LNCap prostate cancer cell line incubated with gossypetin for 24 h, only low concentrations of approximately 25 µmol/L—but not higher or lower—induced autophagy [61]. Under the conditions used in the mentioned study, autophagy was identified through the increased expression of membrane-bound microtubule-associated protein light chain 3 (LC3-II), autophagy-related (Atg)5-Atg12 conjugate, phosphatidylinositide 3 kinase (PI3K), and Beclin-1. In addition to inducing apoptosis and autophagy, in KYSE30 and KYSE510 esophageal cancer cells, gossypetin—20 and 40 µmol/L for 24 h—arrested the cell cycle at the G2/M phase [60], suggesting the additional mechanisms of cancer cell antiproliferative action of this compound.

The MG-63 cell line is known to induce osteoclast formation from mononuclear cells, which is relevant to OS prognosis [62,63,64,65]. Among all OS cell lines, the MG-63 line has been reported to display an increased production of IFN-γ, a cytokine with biphasic effects that contributes to osteoclastogenesis under proinflammatory conditions [66,67]. The results from our study reveal that gossypetin has anti-inflammatory potential in OS, demonstrated by the preventive effect in the production of proinflammatory cytokines in MG-63 cells preincubated with gossypetin and co-incubated with proinflammatory cytokines. The role of proinflammatory cytokines in OS progression and metastization is not completely elucidated. Although the role of flavonoids as modulators of cytokine production is described, only a few reports discuss their role in OS [68]. Zhang et al. observed that, in MG-63 cells, preincubation with the flavonoid avicularin, (quercetin-3-O-α-L-arabinofuranoside)—30 µmol/L for 48 h—counteracted the increased production of IL-6, IL-1β, and TNF induced by a proinflammatory bradykinin stimulus [69]. The observed anti-inflammatory activity was mechanistically linked to the inhibition of the p38MAPK/NF-κB pathway, thus suggesting the potential of this flavonoid in OS therapy. In our study, the stimulation of MG-63 cells with a proinflammatory stimulus (TNF + IL-1β + IFN-γ) enhanced IL-6 and IL-8 production in this cell line. IL-6 and IL-8 have recently been implicated in OS progression and metastasis. Tu et al. observed that IL-6 production by MSCs in the bone microenvironment promoted OS metastization via STAT3 pathway activation in OS cells [17]. Moreover, in murine xenograft models, IL-6 and IL-8 production by OS cells correlated with cancer cell metastatic potential [16]. Importantly, IL-8 production in OS was found to form loops between the OS cells and MSCs, and the administration of anti-IL-8 antibody abrogated these loops and decreased metastization [18]. Regarding IL-6 production, in Swiss albino mice irradiated with γ-radiation, gossypetin pretreatment ameliorated the hepatic function by decreasing oxidative stress and inflammation, including the serum IL-6 levels, revealing an anti-inflammatory action of this compound [70]. In addition to the roles of IL-6 and IL-8, IL-1β also plays a role in OS metastization. In this respect, Han et al. reported that, in MG-63 cells, IL-1β secreted by polarized M2 tumor-associated macrophages (M2-TAMs) induced the expression of several OS metastasis markers via the NF-κB/miR-181α-5p/RASSF1A/Wnt pathway [71]. Moreover, Hu et al. (2017) concluded that IL-1β was significantly upregulated in OS tissues and cell lines, while miR-506 was downregulated [72]. It was observed that upon stimulation with IL-1β in MG-63 cells, NF-κB was activated, and this activation resulted in decreased miR-506 expression, which ultimately increased the expression of Notch protein JAG1, which is involved in OS metastasis. In addition, Jin et al. correlated higher levels of caspase 1 with its downstream target IL-1β in OS tissues compared to peritumoral tissues [73]. In the same study, in MG-63 cells, the isoquinolide alkaloid berberine inhibited caspase 1, which resulted in decreased IL-1β expression. The latter observation was reproduced in BALB/c-nu/nu mice xenografts of MG-63 cells and was confirmed with a caspase 1 inhibitor control. In our study, gossypetin was found to have anti-inflammatory potential in OS as it effectively prevented the production of proinflammatory cytokines in the MG-63 cells when subjected to a proinflammatory cytokine mixture. This is significant because proinflammatory cytokines such as IL-6 and IL-8 have been implicated in OS progression and metastasis. Due to their proinflammatory and immunomodulatory properties, the mixture of proinflammatory cytokines used in this study can potentially activate several cellular pathways, including the NF-κB, JAK-STAT, and MAPK pathways. Additionally, the mixture can potentially activate the inflammasome, with the consequent modulation of PD-L1 expression in cancer cells, which, in the context of a tumor, leads to the recruitment of immune-suppressive cells within the tumor microenvironment. In KBM-5 myeloid leukemia cells and in MDA-MB-231 breast cancer cells, NF-κB activation was not significantly inhibited by gossypetin, thus questioning the role of gossypetin as an NF-κB pathway inhibitor in these cell lines [74,75]. Interestingly, gossypin, differing from gossypetin by a β-D-glucopyranosyl residue attached at position 8, was reported to inhibit NF-κB activation in KBM-5 myeloid leukemia cells under the same conditions reported for gossypetin [75]. Gossypetin has nevertheless been reported to counteract other proinflammatory pathways. For instance, Xie et al. observed that gossypetin inhibited p38 phosphorylation via the direct inhibition of MKK3 and MKK6 phosphorylation in KYSE30 and KYSE410 esophageal cancer cells, highlighting the inhibition of the MAPK pathway in these cell lines [60]. In agreement with these observations, in HaCaT immortalized keratinocytes, gossypetin inhibited PDZ-binding kinase (PBK) phosphorylation, which is involved in the regulation of p38 MAPK and ERK1/2 kinases, thus alleviating the UV-induced phosphorylation of these proteins [28]. In the present study, the ability of gossypetin to mitigate the production of these cytokines in MG-63 cells suggests its potential in countering the inflammation associated with OS, which is possibly linked to MAPK pathway inhibition; however, more research is necessary to elucidate these mechanisms.

The utilization of flavonoids in cancer therapy faces several obstacles that have repercussions for the present study, such as limited potency or efficacy, which may be circumvented by identifying the synergistic relationships with traditional chemotherapeutic medications; additionally, issues with flavonoid stability, specificity, and targeting to cancer cells, which may be addressed with more suitable formulations. Furthermore, these compounds exhibit low water solubility, rapid elimination from the bloodstream, and inadequate bioavailability, which impedes their direct implementation in clinical settings. Further research is therefore necessary to elucidate the precise mechanisms by which flavonoids exert their effects and to optimize their use in the prevention and treatment of osteosarcoma metastasis.

## 5. Conclusions

OS metastization is associated with MSC/tumor cell and immune cell/tumor cell proinflammatory cytokine loops. Regrettably, few studies have addressed the role of flavonoids—compounds with established anti-inflammatory properties—in OS tumor cytokine production. The antiproliferative effects and inhibition of proinflammatory cytokine production observed herein indicate a positive role of gossypetin in therapeutic regimes in osteosarcoma, with potential consequences in the prevention of OS metastasis.

## Figures and Tables

**Table 1:** SAR of inhibition of osteosarcoma cell viability and growth.

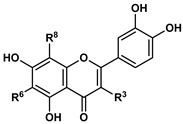				WST-8 assay (IC_50_ ± SEM)				SRB assay (IC_50_ ± SEM)			
No.	R^3^	R^6^	R^8^	MG-63	Saos-2	HOS	143B	MG-63	Saos-2	HOS	143B
**A1**	H	H	H	83 ± 2	67 ± 4	59 ± 2	81 ± 3	67 ± 4	55 ± 6	61 ± 2	61 ± 4
**A2**	OH	H	H	142 ± 8	70 ± 4	72 ± 5	117 ± 10	113 ± 10	61 ± 5	81 ± 4	88 ± 23
**A3**	OH	H	OH	27 ± 2	38 ± 1	12 ± 1	35 ± 6	24 ± 2	30 ± 3	11 ± 1	33 ± 5
**B1**	Cl	H	H	56 ± 6	62 ± 4	60 ± 2	49 ± 5	37 ± 2	37 ± 4	18 ± 2	21 ± 1
**B2**	Cl	H	Cl	128 ± 10	109 ± 6	128 ± 17	80 ± 5	95 ± 2	87 ± 2	31 ± 5	40 ± 3
**B3**	H	Cl	H	93 ± 2	98 ± 5	37 ± 3	49 ± 4	78 ± 9	68 ± 5	34 ± 1	27 ± 2
**B4**	H	Cl	Cl	126 ± 2	116 ± 6	81 ± 8	79 ± 3	127 ± 4	99 ± 8	72 ± 2	64 ± 5
**C1**	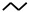	H	H	125 ± 14	84 ± 7	64 ± 7	94 ± 5	79 ± 2	56 ± 7	25 ± 4	37 ± 3
**C2**	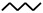	H	H	100 ± 4	74 ± 9	62 ± 1	58 ± 2	75 ± 9	68 ± 4	32 ± 1	37 ± 1

## Data Availability

The data that support the findings of this study are available from the corresponding author upon reasonable request.

## Supplementary Materials

The following supporting information can be downloaded at: https://www.mdpi.com/article/10.3390/antiox12091744/s1, Figure S1: Effects of gossypetin (compound **A3**) in human lung fibroblasts. MRC5 cells were exposed for 48 h to DMSO control or different concentrations of gossypetin (A) effects on fibroblast viability (WST-8 assay); (B) effects on fibroblast growth (SRB assay). The data are mean ± SEM (*n* ≥ 3). Significant differences (asterisks) are shown relative to 0.1% DMSO control; **** *p* < 0.0001 (one-way ANOVA).
