# Supplementary material for: Gossypetin Is a Novel Modulator of Inflammatory Cytokine Production and a Suppressor of Osteosarcoma Cell Growth
Source: Antioxidants (Basel). 2023 Sep 10;12(9):1744. doi: 10.3390/antiox12091744 (PMC10525374; doi:10.3390/antiox12091744)
Supplement: Supplementary file 1 [file antioxidants-12-01744-s001.zip › antioxidants-2569459-supplementary.pdf]

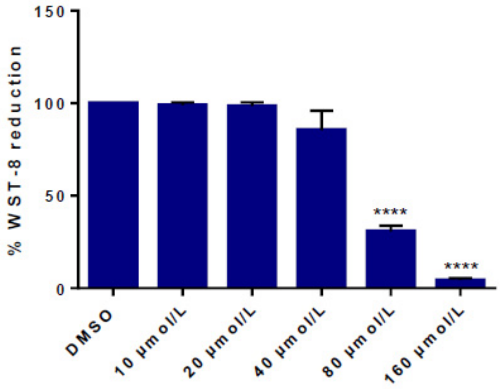

A

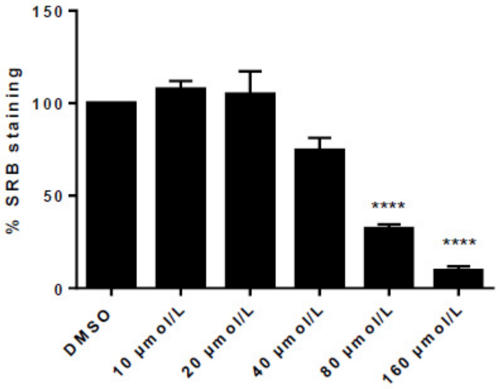

B

Figure S1. Effects of gossypetin (compound A3) on viability and growth of human lung fibroblasts. MRC5 cells were exposed for 48 h to DMSO control or gossypetin at increasing concentrations. (A) effects on fibroblast viability (WST-8 assay); (B) effects on fibroblast growth (SRB assay). The data are mean ± SEM (N≥3). Significant differences (asterisks) are shown relative to 0.1% DMSO control; **** $p < 0.0001$ (one-way ANOVA).
